# Panel Pay and Extended Services—II

**Published:** 1923-06

**Authors:** R. W. Harris


### PANEL PAY AND EXTENDED SERVICES.—II.

By R. W. HARRIS.

IF a panel doctor says to his patient, "You need an X-ray examination, but I cannot give it you," or "I have no proper laboratory facilities, you must go into hospital"; or "An operation would put you right, but it will cost you five guineas"; or "What I am now going to render is a specialist service, beyond the general standard of competence of my fellow-practitioners, and I am therefore under no obligation to give the service—it will cost you two guineas," the panel service is thereby discredited. It matters not that the same thing exactly would happen if the patient were a private patient, and that the insurance patient is getting the full range of family-doctor treatment. He will continue to expect something more, and, until he gets it, to regard the panel service as cheap and nasty. There is no question here of the iniquity of the panel doctor. It is solely the question of an inadequate service, of a service which is "cribb'd, cabin'd and confin'd" because of supposed financial necessities. The terms of the National Insurance Act, 1911, are quite wide enough to embrace a fuller medical service than that at present given, which is limited, by the definition in the Medical Benefit Regulations, to such treatment as can, consistently with the best interests of the patient, properly be undertaken by a general practitioner of ordinary competence and skill. It needs to be emphasised that that limitation is imposed solely because the funds available have not been considered to be adequate to pay for a wider range of service.

### The Present Negotiations.

The Press at the present time is full of mysterious references to the overhauling of the panel system, which is supposed to be under way. Not only are wonderful improvements in course of being effected by some proposal to pay the doctors by the visit, instead of by counting the numbers of persons on their lists, but Minister, Approved Societies, and doctors are all—so we are led to believe——pooling their experience and their proposals to bring about some revolutionary change which, at the end of the present year of grace, will see a reorganised health service for the insured, putting them all henceforth on an A1 in place of a C3 footing. Nothing of the kind is, of course, happening. Dr. Brackenbury has recently told a Liverpool audience that the Society representatives have been through the Regulations and have found comparatively few matters that need altering, if the limitations now imposed on the range of service are to be regarded as unavoidable. Beyond those matters there remains the question of the settlement, once more, under the changing conditions of the time, of the vexed question of the appropriate capitation fee for the insurance practitioner. There is no evidence of any proposal being effectively under consideration for extending the medical service for the insured.

### The Financial Position.

There could be no question at the present time of giving a wider range of medical service to the insured population if it were necessary to look outside National Health Insurance funds for the cost of the extension. But it is not. Sir Thomas Neill, as I have previously indicated, has thrown out a hint of the developments which could be paid for by the sacrifice of one day's sickness benefit all round. But it is more than questionable whether even such a cutting down is necessary in the present healthy state of insurance funds. There were large surpluses disclosed on the valuation of Approved Societies' funds at the end of 1918. There is every indication that further large surpluses will be disclosed on the valuations as at the end of 1923. This means in effect that while, in a national service—financed by compulsory national contributions of employers and employed and by the National Exchequer—there is a defective medical service, because the funds, it is considered, will not allow of a better one, profits are accumulating which pass into the lockers of particular societies to be distributed in due course in a form which will be most popular—in other words, in a majority of cases, frittered away in additional cash payments.

### The Need for a Good Medical Service.

It cannot be gainsaid that it is by the quality of the medical service provided that the success or failure of the National Health Insurance scheme is determined. Everyone wants a good medical service—the Approved Societies are unhesitating in their demands for such a service. Does it not seem folly in these circumstances for a panel doctor to have to address his patient in one or other of the ways which have been suggested at the beginning of this article, even though he may add, by way of solatium, "You will be ill for a good many more weeks than would have been really necessary for your effective recovery if these defects in the medical service were remedied; but, never mind, during the whole of that period your society, which had an excellent valuation, will be able to pay you an extra 2s. 6d. a week?"

### The Specialists are Ready.

A good laboratory service would cost relatively little. The more costly extension, that of bringing the services of the specialist and the operating surgeon within the reach of the insured person, is well worth the risk of the charge on insurance funds which it would involve. The panel doctors' remuneration is a separate question for settlement on its merits, and in the extensions to which I am referring there is no question of putting more money into the pockets of the panel doctors. It is common knowledge that discussions in the time of the late Sir Robert Morant were held with most eminent members of the profession as to the attitude which might be expected towards a development of the insurance medical service so as to embrace operations and other specialist services. The reception given to the very tentative proposals laid before those eminent gentlemen encourages one to think that the specialist services would be forthcoming.

